# Awareness and trust of the FDA and CDC: Results from a national sample of US adults and adolescents

**DOI:** 10.1371/journal.pone.0177546

**Published:** 2017-05-16

**Authors:** Sarah D. Kowitt, Allison M. Schmidt, Anika Hannan, Adam O. Goldstein

**Affiliations:** 1 Department of Health Behavior, Gillings School of Global Public Health, University of North Carolina at Chapel Hill, Chapel Hill, NC, United States of America; 2 Lineberger Comprehensive Cancer Center, University of North Carolina at Chapel Hill, Chapel Hill, NC, United States of America; 3 Department of Family Medicine, University of North Carolina at Chapel Hill, Chapel Hill, NC, United States of America; University of California San Diego, UNITED STATES

## Abstract

Trust in government agencies plays a key role in advancing these organizations' agendas, influencing behaviors, and effectively implementing policies. However, few studies have examined the extent to which individuals are aware of and trust the leading United States agencies devoted to protecting the public’s health. Using two national samples of adolescents (*N* = 1,125) and adults (*N* = 5,014), we examined demographic factors, with a focus on vulnerable groups, as correlates of awareness of and trust in the Centers for Disease Control and Prevention (CDC), Food and Drug Administration (FDA), and the federal government. From nine different weighted and adjusted logistic regression models, we found high levels of awareness of the existence of the FDA and CDC (ranging from 55.7% for adolescents’ awareness of the CDC to 94.3% for adults’ awareness of the FDA) and moderate levels of trust (ranging from a low of 41.8% for adults’ trust in the federal government and a high of 78.8% for adolescents’ trust of the FDA). In the adolescent and adult samples, awareness was higher among non-Hispanic Blacks and respondents with low numeracy. With respect to trust, few consistent demographic differences emerged. Our findings provide novel insights regarding awareness and trust in the federal government and specific United States public health agencies. Our findings suggest groups to whom these agencies may want to selectively communicate to enhance trust and thus facilitate their communication and regulatory agendas.

## Introduction

The Centers for Disease Control and Prevention (CDC) and the Food and Drug Administration (FDA) are two United States (US) federal agencies charged with protecting the public’s health. Both disseminate health information through nationwide campaigns [1] and are expected to serve as reliable and accurate sources of information. The effectiveness of these agencies (as measured by individuals’ responsiveness and compliance with governmental messages and regulations) likely depends on the extent to which these agencies are perceived as trustworthy, competent, and credible [2, 3].

A growing body of research has shown associations between trust in government and health-related behaviors and outcomes [4]. For instance, in a study of HIV-positive adults, individuals with higher trust in government reported greater use of health care services, increased use of antiretroviral medications, fewer emergency room visits, and improved mental and physical health [5]. Other studies have demonstrated the importance of governmental trust with vaccination intentions and uptake [6–9]. From these studies, trust in the government and the health information it communicates can positively impact health outcomes.

Limited research also suggests that trust in government differs by individual-level characteristics. In a 2015 poll from the Pew Research Center, only 19% of Americans reported that they trust the US federal government always or most of the time [10]. Conversely, trust in the FDA and CDC are higher than the federal government, and in the same 2015 poll, 51% and 71% of the US population reported that they view the FDA and CDC favorably, respectively [10]. Individuals that are White [5, 10], have lower incomes, and are older [10–12] tend to be less trusting of the government [11, 12]. For instance, in 2015, 27% of adults between the ages of 18 and 30 said they trusted the federal government at least most of the time, compared with 19% of those aged 30–49 and 15% of those aged 50 and older [10]. However, other studies have shown conflicting results for race and no consistent trends by educational level [11, 12].

No studies to our knowledge have examined trust in governmental agencies among other vulnerable populations. In the current paper, we examine predictors of awareness and trust in two key US health agencies, the CDC and the FDA, using national samples of adolescents and adults. Given associations between age and trust in the government, we hypothesized that trust in the CDC and FDA would be lower among adults compared to adolescents [10–12]. Moreover, given increased knowledge about the government and politics among adults and older adults [13], we hypothesized that adolescents would be less aware of the CDC and FDA than adults.

## Methods

### Sampling

Data utilized in this research come from two separate national phone surveys administered by the Center for Regulatory Research on Tobacco Communication (CRRTC) between September, 2014 and June, 2015 [14]. One survey was administered to adults over the age of 18, and a separate, independent survey was administered to adolescents aged 13–17 years old. Both surveys used random-digit dialing. The survey included questions on tobacco regulatory constructs, such as tobacco regulatory agency credibility. Prior to the survey, consent was required for adults and both assent and parent/guardian consent was required for adolescents. In addition, parents/guardians of adolescents answered some brief demographic questions following the adolescent survey. The adult sample resulted in 5,014 interviews and a weighted response rate of 42%, calculated using American Association for Public Opinion Research (AAPOR) Response Rate 4. The youth sample resulted in 1,125 interviews and a weighted response rate of 66% (also calculated using AAPOR Response Rate 4). In both national samples, results are nationally representative; for the adolescent sample, the weighted sample is nationally representative of 13–17 year olds living in the US with cell or landline access, who could expect to obtain consent from a guardian for a tobacco use phone survey. All procedures were approved by the UNC Chapel Hill Institutional Review Board. For more details on the survey methodology, please refer to Boynton et al., 2016 [14].

### Measures

#### Outcome variables

Outcome variables used in our study included awareness of the CDC and FDA, trust in the CDC and FDA, and trust in the federal government. To assess awareness, two questions were asked of adolescents and adults: “Have you ever heard of the…” followed by an option for the CDC (stated over the phone as “the CDC or the Centers for Disease Control and Prevention”) and an option for the FDA (stated over the phone as “the FDA or Food and Drug Administration”). Individuals who reported having heard of the CDC or the FDA were then asked, “In your opinion, does the (CDC or FDA) give trustworthy information to the public?” For all four items, participants were instructed to respond “yes” or “no”.

Before the main study was conducted, we used cognitive interviewing to determine if adolescents and adults understood these outcome variables; no problems were reported. Since a large proportion of respondents reported uncertainty regarding their trust in the CDC or FDA (ranging from 13% to 21% of adolescents and adults), we also analyzed results with a separate category for individuals who reported uncertainty (i.e., “don’t know” or “not sure”). The results from these multinomial models did not qualitatively differ from the logistic regression in which uncertain responses were combined with “no”. Therefore, since it was of direct interest to examine predictors associated with trust, we combined the uncertain responses with the “no” responses and employed a multivariable logistic regression approach.

For trust in the federal government, only adults were asked, “How much trust do you have in the federal government?”. We categorized responses as “high trust” (i.e., respondents who answered “a great deal” or “a fair amount of trust”) or “low trust” (i.e., respondents who answered “not very much”, “none at all”, or “no opinion”).

#### Education

For education, participants were asked of their highest degree of schooling, with categories created for: “high school degree or less”, and “greater than a high school degree”. For adolescents, this same question was asked directly to parents.

#### Numeracy

Numeracy (i.e., mathematical literacy) has been shown to be associated with medical decision making and evaluations of risk communication [15] and is related to racial and demographic disparities in health outcomes [16]. To assess numeracy, adults and adolescents were asked, “In general, which of these numbers shows the biggest risk of getting a disease?”. Participants who answered “one in 10” (the correct answer) were classified as having high numeracy; all others were classified as having low numeracy [17].

#### Census region

Participants were classified as belonging to one of four census regions: the Northeast, the Midwest, the South, and the West [18].

#### Current cigarette use

Previous research has demonstrated lower levels of trust among cigarette smokers in sources of health information [19]. In addition, both the CDC and FDA communicate about tobacco use, which is the leading cause of preventable death in the US and worldwide [20]. Two of their most prominent and current campaigns are the CDC’s Tips From Former Smokers campaign and the FDA’s The Real Cost. For these reasons, we assessed current cigarette smoking as a predictor of awareness and trust.

Adult participants were asked, “Have you smoked at least 100 cigarettes in your entire life?” and “Do you now smoke cigarettes every day, some days, or not at all?”. Participants who reported smoking at least 100 cigarettes in their lifetime and reported smoking cigarettes every day or some days were classified as current cigarette users. Adolescents who reported ever trying a cigarette and reported using at least one cigarette in the past 30 days were categorized as current cigarette users.

#### Sexual orientation

Adults were asked to identify their sexual orientation and adolescents were asked to describe how they were sexually attracted to other people. For both adults and adolescents, responses for gay, lesbian, and bisexual (“GLB”) were combined.

#### Poverty status

For adults, poverty status was classified as above or below the 2014 poverty line.

### Data analysis

Analyses for this study were conducted with SAS version 9.3 [21]. Sampling weights were used to account for the complex survey design. Descriptive analyses and cross-tabulations were used to generate weighted percentages and confidence intervals of independent and dependent variables. For awareness and trust in CDC, FDA, and the federal government, we conducted nine separate multivariable logistic regression models (4 models for awareness of the CDC and FDA; 5 models for trust of the CDC, FDA, and federal government). In each case, the reference group included individuals who reported not being aware of or not trusting the CDC, FDA, or federal government. Results from the logistic regression models included weighted percentages, adjusted odds ratios (aOR) and confidence intervals (CI). For all analyses, statistical significance was set at p < 0.05.

## Results

### Demographic characteristics

Table 1 provides weighted percentages for our sample of adolescents (*N* = 1,215) and adults (*N* = 5,014). For both samples, approximately half of the participants were female (48.7% and 51.5%), and majority White non-Hispanic (68.9% and 62.1%) and straight or heterosexual (96.1% and 96.8%). Most parents of adolescents reported having a high school degree or greater (80%), as did adults (57.4%). The average ages of adolescents and adults were 15 years and 46.7 years, respectively. Approximately one sixth of adults reported being a current smoker (17.8%), while 3% of adolescents reported being a current smoker. Lastly, approximately one-third of adolescents and adults were classified as having low numeracy (27% and 31.9%).

**Table 1:** Weighted percentages for independent variables from national phone surveys administered by the Center for Regulatory Research on Tobacco Communication in 2014–2015, stratified by adults (*N* = 5,014) and adolescents (*N* = 1,125).

Variable	All adults, n (weighted %) or mean (standard deviation)	All adolescents, n (weighted %) or mean (standard deviation)
Gender		
Female	2372 (48.52)	564 (48.66)
Male	2640 (51.48)	561 (51.34)
Age, continuous	46.73 (0.48)	15.02 (0.04)
Age, dichotomized^a^		
26+ years	4205 (85.1)	
18–25 years	809 (14.9)	
Race		
White non-Hispanic	3280 (62.06)	857 (68.90)
Black non-Hispanic	948 (17.74)	114 (12.52)
Other non-Hispanic	328 (5.90)	68 (8.73)
Hispanic	432 (14.30)	84 (9.84)
Education (or parent’s education^b^)		
More than high school	3241 (57.42)	879 (79.95)
High school or less	1756 (42.58)	244 (20.05)
Sexual Orientation		
Straight or heterosexual	192 (3.2)	42 (3.93)
GLB	4730 (96.8)	1041 (96.07)
Numeracy		
High	3401 (68.09)	818 (72.97)
Low	1599 (31.91)	307 (27.03)
Smoking Status		
Not a current smoker	1151 (17.79)	40 (3.03)
Current smoker	3856 (82.21)	1085 (96.97)
Census Region		
Northeast	537 (18.25)	153 (17.17)
Midwest	972 (21.53)	282 (21.55)
South	2685 (37.11)	550 (37.63)
West	819 (23.11)	139 (23.65)
Poverty Status^c^		
Above poverty line	3772 (82.53)	
Below poverty line	868 (17.47)	

Abbreviation: Gay, Lesbian, or Bisexual (GLB).

^a^ Age was dichotomized for adults only (to compare young adults between the ages of 18–25 and adults over the age of 25).

^b^ Adolescents’ parents were asked to report their education.

^c^ Poverty status was not assessed in the adolescent sample.

**Fig 1:**
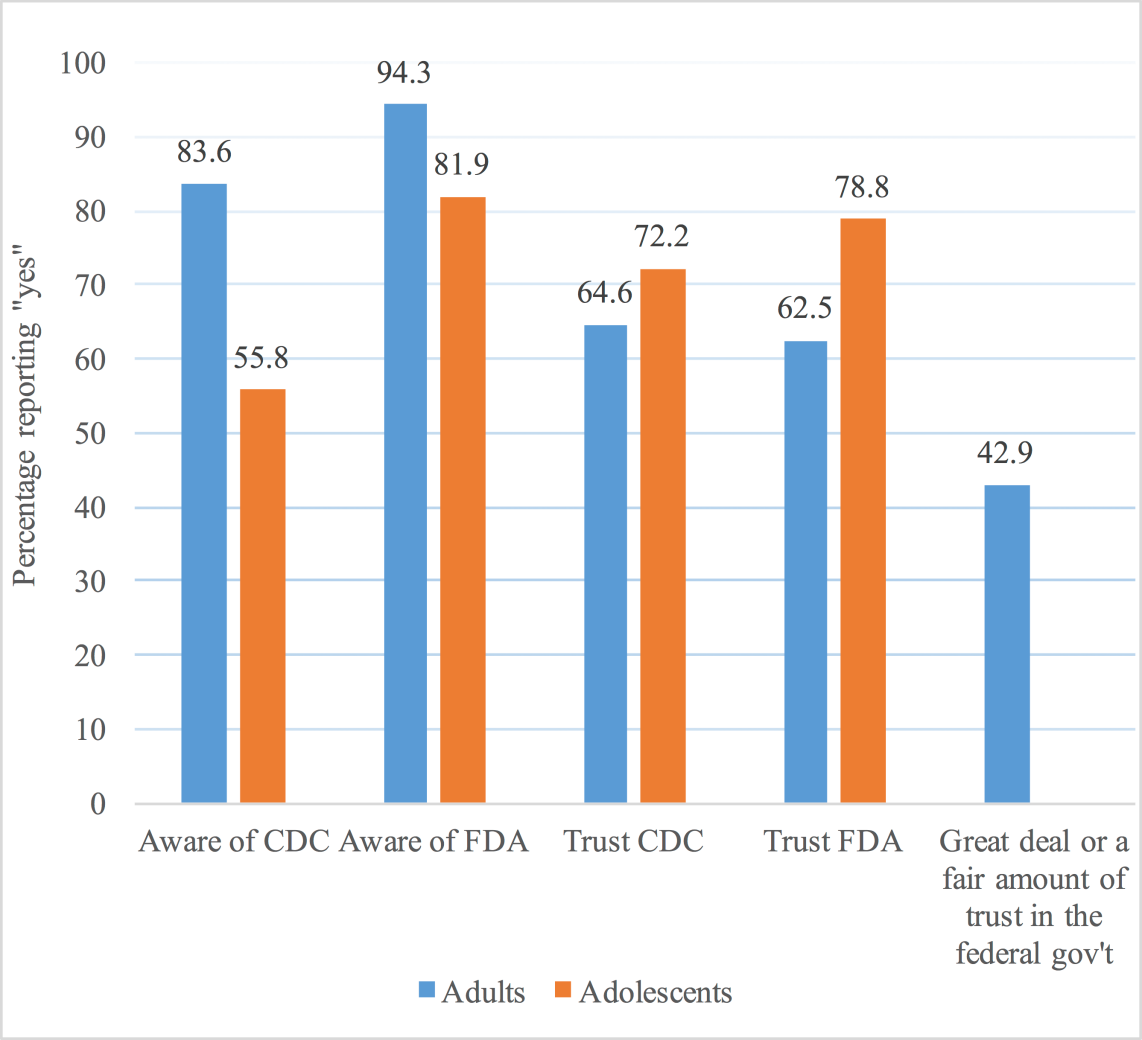
Weighted frequencies of key dependent variables, stratified by adults and youth. Percentage of adults and adolescents who report being aware of or trusting the CDC, FDA, and federal government.

Among adults, awareness of CDC and FDA was high (83.6% and 94.3%, respectively) while moderate awareness existed among adolescents (55.8% and 81.9%, respectively) (Fig 1). Adolescents reported high levels of trust in the CDC and FDA (72.2% reported trusting the CDC and 78.8% reported trusting the FDA), while adults reported moderate levels of trust (64.6% reported trusting the CDC and 62.5% reported trusting the FDA). Lastly, adults reported higher trust in CDC and FDA than the federal government (42.9% reported high trust in the federal government).

### Associations for awareness of the CDC and FDA

A full description of the weighted logistic regression results for awareness of the CDC and FDA can be seen in Table 2 and S1 File. For brevity, key results are summarized below. Across both adolescents and adults, being Black non-Hispanic (compared to White non-Hispanic) and having low numeracy (compared to high numeracy) were significantly associated with lower odds of awareness of the CDC and FDA.

**Table 2:** Weighted logistic regression results comparing participants who reported awareness vs. participants who did not report awareness of the CDC and FDA from national phone surveys administered by the Center for Regulatory research on Tobacco Communication in 2014–2015.

Variable	Adolescents’ Awareness of CDC, *N* = 1,079	Adolescents’ Awareness of FDA, *N* = 1,079	Adults’ Awareness of CDC, *N* = 4,540	Adults’ Awareness of FDA, *N* = 4,540
	aOR (95% CI)	aOR (95% CI)	aOR (95% CI)	aOR (95% CI)
Gender				
Female	REF	REF	REF	REF
Male	1.60 (1.20–2.14)*	1.84 (1.24–2.73)*	0.90 (0.65–1.24)	0.96 (0.53–1.74)
Age, continuous	1.67 (1.50–1.87)*	1.94 (1.65–2.29)*		
Age, dichotomized^a^				
26+ years	REF	REF	REF	REF
18–25 years			0.52 (0.36, 0.75)*	0.42 (0.22, 0.79)*
Race				
White non-Hispanic	REF	REF	REF	REF
Black non-Hispanic	0.45 (0.28–0.71)*	0.39 (0.22–0.67)*	0.44 (0.30–0.65)*	0.27 (0.14–0.55)*
Other non-Hispanic	1.22 (0.70–2.14)	1.14 (0.54–2.41)	0.32 (0.18–0.56)*	0.29 (0.12–0.71)*
Hispanic	1.15 (0.66–2.00)	0.58 (0.29–1.15)	0.21 (0.14–0.31)*	0.14 (0.07–0.30)*
Education (or parent’s education^b^)				
More than high school	REF	REF	REF	REF
High school or less	0.75 (0.53–1.08)	0.45 (0.29–1.15)	0.31 (0.22–0.43)*	0.21 (0.12–0.37)*
Sexual Orientation				
Straight or heterosexual	REF	REF	REF	REF
GLB	2.28 (0.99–5.26)	2.06 (0.46–9.23)	2.15 (1.04–4.44)*	0.49 (0.22–1.10)
Numeracy				
High	REF	REF	REF	REF
Low	0.39 (0.28–0.53)*	0.34 (0.22–0.51)*	0.61 (0.44–0.84)*	0.45 (0.25–0.78)*
Smoking Status				
Not a current smoker	REF	REF	REF	REF
Current smoker	0.57 (0.23–1.41)	3.52 (0.77–16.02)	0.94 (0.65–1.38)	1.11 (0.58–2.13)
Census Region				
Northeast	REF	REF	REF	REF
Midwest	0.63 (0.40–1.01)	0.83 (0.41–1.69)	0.78 (0.44–1.38)	0.35 (0.14–0.84)*
South	0.84 (0.54–1.29)	0.67 (0.35–1.27)	0.98 (0.61–1.58)	0.68 (0.30–1.53)
West	0.33 (0.20–0.57)*	0.35 (0.17–0.74)*	0.83 (0.49–1.39)	0.55 (0.23–1.35)
Poverty Status^c^				
Above poverty line			REF	REF
Below poverty line			0.64 (0.44–0.92)*	0.51 (0.28–0.93)*

Abbreviations: Centers for Disease Control and Prevention (CDC), Food and Drug Administration (FDA), Referent Group (REF), Gay, Lesbian, or Bisexual (GLB), Adjusted Odds Ratio (aOR), Confidence Interval (CI).

^a^ Age was dichotomized for adults only (to compare young adults between the ages of 18–25 and adults over the age of 25).

^b^ Adolescents’ parents were asked to report their education.

^c^ Poverty status was not assessed in the adolescent sample.

* *p* ≤ 05.

For adolescents only, greater odds of awareness in the CDC and FDA occurred for males, with increasing age, and adolescents living in the South, compared to adolescents living in the Northeast. For adults only, lower odds of awareness of the CDC and FDA occurred for Hispanic and other non-Hispanic adults, adults with a high school degree or less, adults whose income fell below the poverty line, and young adults between the ages of 18 and 25, compared to their counterparts. Lastly, adults identifying as GLB had greater odds of awareness of the CDC, than adults identifying as straight or heterosexual.

### Associations for trust in the CDC and FDA

A full description of the weighted logistic regression results for trust in the CDC and FDA among adolescents and adults can be seen in Table 3 and S2 File. Overall, few covariates were consistently associated with trust across adolescents and adults. Among adolescents, identifying as Black non-Hispanic was associated with lower odds of trusting the CDC than identifying as White non-Hispanic. For adolescents’ trust in the FDA, individuals of older ages showed higher odds of trusting the FDA; conversely, individuals identifying as Hispanic (compared to non-Hispanic White individuals) and current smokers showed lower odds of trusting the FDA.

**Table 3:** Weighted logistic regression results comparing participants who reported trust vs. participants who did not report trust of the CDC, FDA, and federal government from national phone surveys administered by the Center for Regulatory research on Tobacco Communication in 2014–2015^d^.

Variable	Adolescents’ Trust in CDC, *N* = 632	Adolescents’ Trust in FDA, *N* = 905	Adults’ Trust in CDC, *N* = 3,867	Adults’ Trust in FDA, *N* = 4326	Adults’ Trust in Federal Gov’t, *N* = 4526
	aOR (95% CI)	aOR (95% CI)	aOR (95% CI)	aOR (95% CI)	aOR (95% CI)
Gender					
Female	REF	REF	REF	REF	REF
Male	1.19 (0.80–1.77)	1.00 (0.70–1.44)	1.12 (0.87–1.43)	1.00 (0.80–1.25)	1.32 (1.06–1.65)*
Age, continuous	1.04 (0.90–1.19)	1.17 (1.02–1.35)*			
Age, dichotomized^a^					
26+ years			REF	REF	REF
18–25 years			1.46 (1.02, 2.09)*	1.25 (0.92, 1.68)	0.83 (0.63, 1.09)
Race					
White non-Hispanic	REF	REF	REF	REF	REF
Black non-Hispanic	0.51 (0.27–0.96)*	0.77 (0.41–1.45)	0.69 (0.48–0.99)*	0.76 (0.55–1.05)	1.59 (1.17–2.15)*
Other non-Hispanic	0.64 (0.30–1.35)	1.05 (0.49–2.27)	0.78 (0.51–1.20)	0.88 (0.59–1.31)	1.53 (1.05–2.23)*
Hispanic	1.00 (0.47–2.15)	0.46 (0.24–0.86)*	0.71 (0.48–1.04)	0.79 (0.55–1.13)	1.27 (0.90–1.78)
Education (or parent’s education^b^)					
More than high school	REF	REF	REF	REF	REF
High school or less	0.79 (0.50–1.25)	0.83 (0.52–1.31)	0.64 (0.48–0.86)*	0.96 (0.74–1.25)	0.83 (0.66–1.06)
Sexual Orientation					
Straight or heterosexual	REF	REF	REF	REF	REF
GLB	1.19 (0.44–3.21)	0.78 (0.33–1.82)	1.24 (0.72–2.15)	1.06 (0.64–1.73)	1.22 (0.78–1.89)
Numeracy					
High	REF	REF	REF	REF	REF
Low	1.31 (0.77–2.23)	0.89 (0.58–1.37)	0.87 (0.68–1.14)	0.86 (0.68–1.09)	0.84 (0.66–1.06)
Smoking Status					
Not a current smoker	REF	REF	REF	REF	REF
Current smoker	0.45 (0.17–1.16)	0.30 (0.14–0.64)*	0.68 (0.51–0.92)*	0.81 (0.62–1.06)	0.52 (0.40–0.68)*
Census Region					
Northeast	REF	REF	REF	REF	REF
Midwest	0.90 (0.49–1.66)	1.26 (0.71–2.23)	0.86 (0.56–1.32)	0.95 (0.65–1.40)	0.81 (0.56–1.18)
South	0.82 (0.47–1.42)	1.13 (0.66–1.88)	0.92 (0.64–1.33)	0.96 (0.69–1.34)	0.82 (0.59–1.13)
West	0.84 (0.40–1.75)	1.27 (0.65–2.50)	0.96 (0.65–1.42)	0.88 (0.62–1.25)	0.82 (0.58–1.15)
Poverty Status^c^					
Above poverty line			REF	REF	REF
Below poverty line			1.42 (0.96–2.11)	1.40 (1.02–1.93)*	1.30 (0.93–1.79)

Abbreviations: Centers for Disease Control and Prevention (CDC), Food and Drug Administration (FDA), Referent Group (REF), Gay, Lesbian, or Bisexual (GLB), Adjusted Odds Ratio (aOR), Confidence Interval (CI).

^a^ Age was dichotomized for adults only (to compare young adults between the ages of 18–25 and adults over the age of 25).

^b^ Adolescents’ parents were asked to report their education.

^c^ Poverty status was not assessed in the adolescent sample.

^d^ Only participants who reported that they were aware of the CDC or FDA were asked questions to assess if they found these agencies to be trustworthy. For this reason, sample sizes are lower and more variable.

* *p* ≤ 05.

For adults’ trust in the CDC, individuals identifying as Black non-Hispanic showed lower odds of trusting the CDC compared to individuals identifying as White non-Hispanic. Additionally, adults with a high school degree or less and current smokers had lower odds of trusting the CDC, while young adults between the ages of 18 and 25 showed greater odds of trusting the CDC, compared to adults over the age of 25. Lastly, for adults’ trust in the FDA only, adults whose income fell below the poverty line had higher odds of trusting agency than adults whose income fell above the poverty line.

### Associations for trust in federal government

A full description of the logistic regression results for trust in the federal government among adults can be seen in Table 3 and S3 File. Men, Black non-Hispanic, and other non-Hispanic adults (compared to White non-Hispanic adults) had higher odds of trusting the federal government; current smokers had lower odds of trusting the federal government.

## Discussion

This is the first nationally representative study examining awareness and trust of the FDA and CDC among adolescents and adults. We found high awareness of CDC and FDA among adults and moderate awareness among adolescents. Conversely, we found high levels of trust in the CDC and FDA among adolescents, but only moderate levels of trust in the CDC and FDA and even lower levels of trust in the federal government among adults. We also found that demographic factors predicted awareness (and to a lesser extent, trust) of the CDC and FDA.

Previous research has established the importance of trust in the government as a predictor of a wide variety of health behaviors and outcomes [4–9]. For instance, during the 2009 H1N1 (i.e., “swine flu”) outbreak, individuals who reported less confidence in the government were less likely to take the vaccine, which resulted in a large amount of unused vaccines [12]. Additionally, in a study of HPV vaccine acceptance, mothers who reported high trust in the government were more likely to accept the vaccine [22]. Moreover, in a recent study examining public support for increasing the minimum age of tobacco product sales, Lee and colleagues found that trust in government was significantly associated with policy support, after controlling for a wide variety of demographic factors [23]. Given the association of trust in government with several different health outcomes, continued research regarding trust in government is important.

Few studies have looked specifically at trust in the CDC and FDA. Using nationally representative data, we found moderate to high levels of trust in the CDC and FDA (ranging from a low of 62% for adult trust in FDA to a high of 79% for adolescent trust in the FDA), which is fairly consistent with a Pew Research study finding 65% of adults trust the FDA and 75% of adults trust the CDC) [24]. We also found that few sociodemographic variables were consistently significantly associated with trust in the CDC or FDA. Given that previous research has shown associations between trust in government and health behaviors and outcomes—including vaccine use, health care utilization, and mental and physical health [4–9]—our finding of consistent reports of trust in CDC, FDA, and the federal government among different vulnerable populations is promising. However, these agencies could seek to increase their trust among groups who are less likely to trust them. For instance, smokers, individuals of lower education, adults (compared to adolescents), and racial and ethnic minorities all might benefit from communications from the CDC or FDA that highlight the significant work these agencies are doing to protect public health for vulnerable groups. It is feasible (and perhaps likely) that individuals who do not trust the CDC or FDA or who are unware of the CDC or FDA may be less likely to engage in preventive services (e.g., cancer screenings, flu shots) or follow recommendations put forth by these agencies [25]. One prior study found that parents who trusted FDA recommendations against administering over-the-counter cold medicines to young children were more likely to follow these recommendations to protect the health of their children [25]. Moreover, these issues may be more pronounced for adolescents or vulnerable populations. For instance, youth who do not trust the FDA may be less likely to believe campaign messages from the Real Cost about smoking. Or African American women who do not trust the CDC or information from the CDC may be less likely to believe that they should take actions to lower their breast or ovarian cancer risk.

A growing body of literature suggests that agencies can use several approaches to increase their level of trust, including faster responses to public health issues, greater transparency, and collaboration between different public health agencies. For example, in the 2014 Ebola outbreak, perceptions of the CDC’s response as slow and inconsistent, in conjunction with distinct health policies adopted by the state and federal governments, diminished adults’ confidence in the CDC and state public health agencies [26]. In another case, the FDA and CDC released conflicting statements and recommendations to the public about the use and efficacy of Tamiflu, a flu medication. As both the FDA and CDC are federal agencies, and presumed to present a united front on health issues, the contrasting messages diminished the public’s trust in both agencies [27]. On the other hand, after the 2001, anthrax outbreak, individuals reported high public trust in the CDC and its public health officials, thereby demonstrating that trust in governmental agencies can also be enhanced [28].

Almost no studies to our knowledge have examined national awareness of the CDC or FDA. Specifically, our findings suggest 1) high awareness of the CDC and FDA among adults and moderate awareness among adolescents and 2) that younger individuals, African Americans, adults with lower reported education, and individuals with low numeracy were less aware of the CDC and FDA compared to their counterparts. One study examining racial differences in health numeracy found that Whites, African Americans, and Hispanics with low health numeracy were less likely to be aware of CDC than those with high health numeracy [29]. In the same study, awareness of the CDC was one of the most important factors influencing health disparities between Whites and Hispanics in terms of health numeracy [29]. Similarly, in our study, we found that adolescents and adults with low numeracy were consistently less likely to be aware of CDC and FDA than those with high numeracy, independent of and in addition to racial / ethnic minorities and individuals with lower education. These findings point to a segment of the population to target to increase awareness (and hopefully subsequently trust) for the missions of the FDA and CDC.

Moreover, as expected, we found high awareness of the CDC and FDA among adults, but only moderate trust in the CDC, FDA, and federal government, compared to adolescents. This is consistent with some past research that has found greater trust in federal agencies among younger age groups [24]. There are several reasons why adults and older adults in particular may have lower trust in the government and governmental institutions. In a study examining Australians’ trust in federal, state, and local government, researchers found that as age increased, respondents were less likely to trust both state and federal government, with the lowest level of trust among respondents aged 60+ [30]. The authors of this study suggested that older Australians were more disadvantaged in terms of job opportunities, were subjected to ageism, and had limited access to public services, all of which could be linked to government initiatives [30]. Older adults may also be more likely to consume media containing critical information about the government [30]. In the US, confidence in government institutions is also influenced by generation status (in addition to age). Baby boomers (born between 1946 and 1964, aged in their 50 to 67 years old in this study) consistently have the lowest amount of trust in the government, compared to those born before and after [31]. Researchers have argued that this phenomenon may be linked to economic conditions (i.e., rising income inequality), which can negatively affect social capital and trust in government institutions [31]. Future research could tease apart the extent to which trust differences between younger and older adults are due to a cohort effects, period effects, or age effects, as there is some evidence for each of these explanations.

Finally, we have reason to believe that trust in the CDC and FDA are important variables for future studies to examine. Literature on source credibility suggests that messages with more credible sources are more likely to be impactful than messages with non-credible sources (e.g., tobacco companies) or no source [32]. In an experiment manipulating message source and content, researchers found that source was an influential variable in determining perceived effectiveness for messages about health consequences of smoking [33]. Moreover, they found that perceptions of credibility mediated the effect of source on ad perceived effectiveness for some sub-populations (e.g., nonsmokers, but not smokers). From these findings, we may expect that trust in the CDC or FDA may influence the relative success of public health campaigns; however, it is also plausible that these patterns may differ for vulnerable populations, for whom we found lower trust of CDC and FDA. Further research exploring how trust in the CDC and FDA may be associated with health outcomes and/or effectiveness of public health ad campaigns is warranted. Moreover, specifically among adolescents, future research could examine the discrepancy between awareness of the FDA (which was high at 82%) and awareness of the CDC (which was moderate at 56%). It is possible that higher awareness of the FDA resulted from specific FDA media campaigns aimed at youth (i.e., “The Real Cost”) [34, 35]. Qualitative research may be useful in exploring this research area.

### Limitations

Our study had several limitations, which suggest directions for future research. Our findings that few variables consistently predicted trust may be due in part to the fact that the trust questions were only asked of people who were aware of the agencies; thus, responses may have been more uniform in this reduced sample than would otherwise be found. It is possible and even likely that individuals who are unaware of the FDA or CDC may not trust these organizations or have uncertainty regarding their trust in these organizations. To fully understand the influences on awareness and trust of government agencies, other influences beyond individual-level predictors should be examined, including organizational factors (e.g. agency transparency), media framing of government actions, and political climate.

## Conclusions

Some of the key public health issues of our time—vaccines, medical devices, pharmaceutical access and uptake, food safety, bioterrorism, tobacco use—are all issues for which trust in the sources of messages about these issues is important. Without trust in the government or federal agencies, public confidence in government communication about the above issues may be diminished. Understanding the factors that affect public trust in the federal government and public health agencies is critical for dissemination of effective health messages [11, 36]. As new research on FDA credibility is forthcoming [32], further investigation of how credibility and trust of federal agencies influence health behaviors and outcomes is needed.

## Supporting information

S1 File Awareness of the CDC and FDA: Interpretations of adjusted odds ratios (Table 2). (DOCX)

S2 File Trust in the CDC and FDA: Interpretations of adjusted odds ratios (Table 3). (DOCX)

S3 File Trust in the Federal Government: Interpretations of adjusted odds ratios (Table 3). (DOCX)
