# Supplementary material for: Awareness and trust of the FDA and CDC: Results from a national sample of US adults and adolescents
Source: PLoS One. 2017 May 16;12(5):e0177546. doi: 10.1371/journal.pone.0177546 (PMC5433718; doi:10.1371/journal.pone.0177546)
Supplement: S1 File — (DOCX) [file pone.0177546.s001.docx]

**S1 File. Awareness of the CDC and FDA: Interpretations of Adjusted Odds Ratios (Table 2).**

*For adolescent awareness of CDC,* greater odds of awareness occurred for increasing age (aOR: 1.67, 95% CI:1.50, 1.87) and being male rather than female (aOR: 1.60, 95% CI: 1.20, 2.14). Lower odds of awareness occurred for adolescents who identified as Black non-Hispanic rather than White non-Hispanic (aOR: 0.45, 95% CI: 0.28, 0.71); adolescents who were classified as having low numeracy (aOR: 0.39, 95% CI: 0.28, 0.53); and adolescents living in the West (aOR: 0.33, 95% CI: 0.20, 0.57) compared to the Northeast.

*For adolescent awareness of FDA*, greater odds of awareness occurred for increasing age (aOR: 1.94, 95% CI: 1.65, 2.29) and being male rather than female (aOR: 1.84, 95% CI: 1.24, 2.73). Lower odds of awareness occurred for adolescents who identified as Black non-Hispanic rather than White non-Hispanic (aOR:0.39, 95% CI: 0.22, 0.67); adolescents who were classified as having low numeracy (aOR: 0.34, 95% CI: 0.22, 0.51); and adolescents who lived in the West compared to the Northeast (aOR: 0.35, 95% CI: 0.17, 0.74).

*For adult awareness of CDC,* greater odds of awareness occurred for GLB adults (aOR: 2.15, 95% CI: 1.04, 4.44), compared to straight or heterosexual adults. Lower odds of awareness for adults occurred for adults who identified as Black non-Hispanic (aOR: 0.44, 95% CI: 0.30, 0.65), other non-Hispanic (0.32, 95% CI: 0.18, 0.56), and Hispanic (aOR: 0.21, 95% CI: 0.14, 0.31), compared to White non-Hispanic; adults who reported a high school degree or less (aOR: 0.31, 95% CI: 0.22, 0.43); adults who were classified as having low numeracy (aOR: 0.61, 95% CI: 0.44, 0.84); adults whose income fell below the poverty line (aOR: 0.64, 95% CI: 0.44, 0.92); and young adults between the age of 18 and 25 (aOR: 0.52, 95% CI: 0.36, 0.75), compared to adults over the age of 25.

*For adult awareness of FDA*, lower odds of awareness occurred for adults who identified as Black non-Hispanic (aOR: 0.27, 95% CI: 0.14, 0.55), other non-Hispanic (aOR: 0.29, 95% CI: 0.12, 0.71), or Hispanic (aOR: 0.14, 95% CI: 0.07, 0.30) compared to White non-Hispanic; adults with a high school degree or less (aOR: 0.21, 95% CI: 0.12, 0.37) compared to adults with greater than a high school degree; adults who were classified as having low numeracy (aOR: 0.45, 95% CI: 0.25, 0.78); adults who lived in the Midwest (aOR: 0.35, 95% CI: 0.14, 0.84); adults whose income fell below the poverty line (aOR: 0.51, 95% CI: 0.28, 0.93); and young adults between the age of 18 and 25 (aOR: 0.42, 95% CI: 0.22, 0.79), compared to adults over the age of 25.
