# Supplementary material for: Awareness and trust of the FDA and CDC: Results from a national sample of US adults and adolescents
Source: PLoS One. 2017 May 16;12(5):e0177546. doi: 10.1371/journal.pone.0177546 (PMC5433718; doi:10.1371/journal.pone.0177546)
Supplement: S2 File — (DOCX) [file pone.0177546.s002.docx]

**S2 File. Trust in the CDC and FDA: Interpretations of Adjusted Odds Ratios (Table 3).**

*For adolescent trust in the CDC*, lower odds of trust occurred for individuals who identified as Black non-Hispanic (aOR: 0.51, 95% CI: 0.27, 0.96), compared to individuals who identified as White non-Hispanic.

*For adolescent trust in the FDA,* greater odds of trust occurred with increasing age (aOR: 1.17, 95% CI: 1.02, 1.35). Lower odds of trust occurred for adolescents who identified as Hispanic (aOR: 0.46, 0.24, 0.86) compared to adolescents who identified as White non-Hispanic and for current smokers (aOR: 0.30, 95% CI: 0.14, 0.64) compared to non-smokers.

*For adult trust in the CDC*, greater odds of trust occurred for young adults between the ages of 18 and 25 (aOR: 2.46, 95% CI: 1.02, 2.09), compared to adults over the age of 25. Lower odds of trust occurred for adults who identified as Black non-Hispanic (aOR: 0.69, 95% CI: 0.48, 0.98), compared to White non-Hispanic; adults with a high school degree or less (aOR: 0.64, 95% CI: 0.48, 0.86), compared to adults with greater than a high school degree; and current smokers (aOR: 0.68, 95% CI: 0.51, 0.92).

*For adult trust in the FDA*, greater odds of trust occurred for adults whose income fell below the poverty line (aOR: 1.40, 95% CI: 1.02, 1.93).
