# Supplementary material for: Awareness and trust of the FDA and CDC: Results from a national sample of US adults and adolescents
Source: PLoS One. 2017 May 16;12(5):e0177546. doi: 10.1371/journal.pone.0177546 (PMC5433718; doi:10.1371/journal.pone.0177546)
Supplement: S3 File — (DOCX) [file pone.0177546.s003.docx]

**S3 File. Trust in the Federal Government: Interpretations of Adjusted Odds Ratios (Table 3).**

*For adult trust in the federal government*, greater odds of trust occurred for adults who were male (aOR: 1.33, 95% CI: 1.06, 1.65); adults who identified as Black non-Hispanic (aOR: 1.59; 95% CI: 1.17, 2.15) and adults who identified as other non-Hispanic (aOR: 1.53, 95% CI: 1.05, 2.23), compared to adults who identified as White non-Hispanic. Lower odds of trust occurred for current smokers (aOR: 0.52; 95% CI: 0.40, 0.68) compared to non-smokers.
